# Robotic surgery: getting the evidence right

**DOI:** 10.5694/mja2.51726

**Published:** 2022-10-02

**Authors:** Wei Shen Tan, Anthony Ta, John D Kelly

**Affiliations:** ^1^ University College London Hospitals NHS Foundation Trust London UK; ^2^ Division of Surgery and Interventional Science University College London London UK

**Keywords:** Surgery, computer‐assisted, Evidence‐based medicine, Randomized controlled trial as topic

Obtaining high quality evidence relating to adoption of robotic surgery presents challenges

Surgery performed using a robotic platform has led to a step change in how some procedures, largely urological, are delivered. Robotic systems are perceived to be advantageous over laparoscopic and open surgery by providing stereoscopic 3D vision with magnification of the surgical field and precise controlled instrument movement to allow dissection in confined spaces and complex manoeuvres such as suturing.

That said, the global adoption of robotic surgery in other specialities has not been at pace, despite what seem to be clear benefits for patients undergoing some complex pelvic and abdominal procedures. Approximately 1.2 million robotic procedures had been performed worldwide as of December 2020, most of which were robotic‐assisted radical prostatectomies (RARPs).^1^ By contrast, many colorectal and gynaecological procedures remain within the remit of laparoscopic or traditional open surgery. In this article, we discuss the evidence and controversy relating to adoption of robotic surgery, challenges in obtaining high quality evidence, and future prospects in implementing new surgical technologies.

Opponents of robotic surgery often cite the lack of evidence to support its use and highlight the high health care cost. In Australia, the cost of the da Vinci Xi (Intuitive Surgical, Inc.) platform is an estimated $3.9 million in addition to consumable costs of $1848 per operation and a service cost of $621 245 for a 3‐year contract.^2^ Intuitive has enjoyed a monopoly, although the introduction of new robotic systems into the market has the potential to alter the health economic landscape.^3^ As competition from newer robotic systems drives cost down, it is conceivable that robust evidence will be important in overcoming barriers to adoption.

Gathering high quality evidence for surgical technology is challenging, and perceived benefits, despite little or no evidence of benefit, are often enough for both patients and surgeons to select a new technology.^4^, ^5^ This is a world‐wide challenge affecting surgical trials more so than non‐surgical trials.^6^, ^7^ Qualitative analysis of the BOLERO trial, a randomised controlled trial (RCT) of open versus minimally invasive cystectomy, reported that most patients declined trial participation because they had a preference for a particular treatment arm, typically the novel treatment.^7^ Similarly, an analysis of patients undergoing radical prostatectomy in England between 2010 and 2014 suggested that men were attracted to centres offering RARP and would bypass centres without a robotic service.^8^

Often, key opinion leaders are early adopters and become advocates for new technologies, which drives expansion and influences decisions before attainment of safety and efficacy data. However, in a catch‐22 position, having gained this new expertise, the momentum to pursue clinical trials is often lacking and lags behind clinical expansion. RARP, first described in 2002, is now the standard of care in most developed countries despite little evidence for benefit.^9^ It was not until 2016, that the first well designed RCT reported outcomes.^10^

An Australian RCT compared RARP to open radical prostatectomy with a primary endpoint of urinary and sexual function at 6 weeks, 12 weeks and 24 months, suggesting they were comparable.^11^ However, analysis of secondary endpoints suggests that RARP affords shorter hospital length of stay (1.6 days *v* 3.3 days), less operative blood loss (443 mL *v* 1338 mL), less intraoperative adverse events (2% *v* 8%), as well as shorter operating time (202 minutes *v* 234 minutes).^11^ While no observed benefit was reported in the primary endpoint of the trial, it did highlight benefits of RARP in terms of earlier patient recovery.

In the LAP‐1 trial, a phase 3 RCT, patients with localised prostate cancer were randomised to either RARP or laparoscopic radical prostatectomy.^12^ In 2021, the trial reported that early continence (the primary endpoint) was better in the RARP arm. A secondary outcome suggested that patients who received a nerve‐sparing procedure had better potency. Specifically, early continence (3 months; *P* = 0.027) significantly favoured RARP with an 9% absolute improvement which was subsequently not significant at 6 months (*P* = 0.68) and 12 months (*P* = 0.38).^12^ Patients who underwent a nerve‐sparing approach had significantly better erections postoperatively at 3 months (*P* = 0.005), 6 months (*P* = 0.018) and 12 months (*P* = 0.013).^12^

It is laudable that these trials were conducted but neither study altered the position of RARP as the standard of care. An argument that non‐randomised data would enable a more real‐time read out and may be sufficient to inform decision making seems attractive; however, without a control arm, it is impossible to make robust comparisons. A comparison of population‐based observational studies and RCTs found no agreement beyond what was expected by chance, indicating that RCTs remain essential.^13^

Evidence purporting to show a benefit for robotic surgery has not been forthcoming to other surgical speciality procedures. In the ROLARR trial, an RCT of 471 patients comparing robotic‐assisted surgery with laparoscopic surgery for rectal cancer, there was no reported difference in risk of conversion to open laparotomy between treatment arms (8% robotic *v* 12% laparoscopic).^14^ Similarly, other endpoints such as perioperative complication rate, 30‐day mortality, bladder and sexual dysfunction, and hospital length of stay were similar.^14^ Randomised trials in less complex procedures such as robotic inguinal hernia and ventral hernia repair reported no observed perioperative benefit but a longer operating time and increased cost compared with laparoscopic surgery.^15^, ^16^

In the case of radical hysterectomy for early‐stage cervical cancer, a robotic‐assisted procedure may result in inferior oncological outcomes.^17^ An RCT of 631 patients comparing minimally invasive hysterectomy (including robotic‐assisted) with an open approach reported that the minimally invasive arm resulted in lower disease‐free survival and overall survival.^17^ This resulted in a change in international guidelines in favour of open hysterectomy and has led to a substantial reduction in minimally invasive surgery for cervical cancer, particularly in United States academic centres.^18^

Uptake of robotic cystectomy continues to lag compared with RARP. The RAZOR trial, a multicentre trial randomising between open radical cystectomy (ORC) and extracorporeal robotic‐assisted radical cystectomy (RARC) concluded that there was no significant difference in 2‐year disease‐free progression (the primary endpoint) between treatment arms (extracorporeal RARC, 72% *v* ORC, 72%).^19^ Secondary perioperative endpoints such as postoperative adverse events were similar, but significantly lower intraoperative blood loss (300 mL *v* 700 mL) and perioperative transfusion rate (24% *v* 45%), and a significantly lower but not clinically meaningful length of stay (6 days *v* 7 days) were reported, favouring extracorporeal RARC. These findings were consistent with previous published meta‐analyses.^19^, ^20^ The RAZOR trial results were contrary to an initial report from an underpowered RCT which reported a greater number of pelvic and abdominal sites of metastatic sites in the robotic arm.^21^ The trial did not recruit fully, and these preliminary findings may have influenced decisions to adopt robotic cystectomy as an alternative to open surgery.

In 2016, National Health Service (NHS) England reviewed evidence for cystectomy and planned to halt the expansion of new robotic cystectomy centres while implementing an evidence‐gathering exercise to obtain real‐world data on the benefits of robotic cystectomy.^22^ If high quality evidence cannot be procured, such an approach could have a significant impact on whether or not robotic RARC receives funding in the United Kingdom. This led to the development of the iROC trial, a multicentre RCT comparing ORC with intracorporeal RARC. This phase 3 trial used a novel primary endpoint of number of days alive and out of hospital within 90 days from surgery,^23^ and has recently reported outcomes.^24^

The challenge here lay in the fact that at our institution, over 95% of all radical cystectomies were performed robotically before the trial commencement. We made the decision to withdraw intracorporeal RARC as our standard cystectomy approach, citing lack of evidence to support its use. Patients were only allowed to have intracorporeal RARC within a trial setting, in contrast to the extracorporeal urinary diversion performed in the RAZOR trial. This resulted in rapid accrual of the required 320 patients from nine UK sites within 36 months.

There are now data to support the use of robotic surgery for radical cystectomy.^24^ The primary outcome of the iROC trial was that patients treated with intracorporeal RARC had a higher number of days out of hospital within 90 days (82 days *v* 80 days). Secondary findings suggested a lower risk of thromboembolic complications (2% *v* 8%) and lower wound complications (6% *v* 16%) in the RARC treated patients.^24^ Patients treated with ORC had poorer quality of life and greater disability at 5 weeks, which was subsequently not significantly different beyond 12 weeks.^24^ All patients were followed up for a minimum of 12 months. Obtaining such evidence might not be possible without the pragmatic approach in trial design and buy‐in from surgeons which improved patient recruitment.

Surgeons remain the main obstacle to the success of surgical randomised trials.^25^ In our pursuit of high quality evidence, we owe it to our patients to set aside personal views, acknowledge that limited evidence is available in certain areas of surgical practice, and support surgical trial recruitment. New technologies should be evaluated in a prompt manner before widespread dissemination, in accordance with IDEAL recommendations (https://www.ideal‐collaboration.net/the‐ideal‐framework/recommendations/), a framework for the assessment of new surgical technology which encompasses the following phases: idea, development, exploration, assessment and long term study.^26^ Evaluating new technologies in an evidence‐based approach in collaborative centralised health networks within surgical technology hubs may aid rapid patient recruitment, particularly in complex and uncommon surgical procedures. This could then enable prompt trial completion before adoption of such technologies and before they are entrenched as standard of care.^27^

## Competing interests

No relevant disclosures.

## Provenance

Commissioned; externally peer reviewed.

## References

[1] Intuitive Surgical Inc. Annual report 2020 . https://isrg.intuitive.com/static‐files/80b10bf5‐c1da‐4ad3‐bb0e‐8c595e2c712c (viewed Aug 2022).

[2] McBride K , Steffens D , Stanislaus C , et al. Detailed cost of robotic‐assisted surgery in the Australian public health sector: from implementation to a multi‐specialty caseload. BMC Health Serv Res 2021; 21: 108. 33522941 10.1186/s12913-021-06105-z PMC7849115

[3] Crew B. Worth the cost? A closer look at the da Vinci robot’s impact on prostate cancer surgery. Nature 2020; 580: S5‐S7.

[4] McCulloch P , Feinberg J , Philippou Y , et al. Progress in clinical research in surgery and IDEAL. Lancet 2018; 392: 88‐94. 29361334 10.1016/S0140-6736(18)30102-8

[5] Angelos P. Ethics and surgical innovation: challenges to the professionalism of surgeons. Int J Surg 2013; 11: S2‐S5. 24380544 10.1016/S1743-9191(13)60003-5

[6] Mouw TJ , Hong SW , Sarwar S , et al. Discontinuation of surgical versus nonsurgical clinical trials: an analysis of 88,498 trials. J Surg Res 2018; 227: 151‐157. 29804847 10.1016/j.jss.2018.02.039

[7] Harrop E , Kelly J , Griffiths G , et al. Why do patients decline surgical trials? Findings from a qualitative interview study embedded in the Cancer Research UK BOLERO trial (Bladder cancer: Open versus Lapararoscopic or RObotic cystectomy). Trials 2016; 17: 35. 26787177 10.1186/s13063-016-1173-z PMC4719666

[8] Aggarwal A , Lewis D , Mason M , et al. Effect of patient choice and hospital competition on service configuration and technology adoption within cancer surgery: a national, population‐based study. Lancet Oncol 2017; 18: 1445‐1453. 28986012 10.1016/S1470-2045(17)30572-7 PMC5666166

[9] Menon M , Shrivastava A , Tewari A , et al. Laparoscopic and robot assisted radical prostatectomy: establishment of a structured program and preliminary analysis of outcomes. J Urol 2002; 168: 945‐949. 12187196 10.1016/S0022-5347(05)64548-X

[10] Yaxley JW , Coughlin GD , Chambers SK , et al. Robot‐assisted laparoscopic prostatectomy versus open radical retropubic prostatectomy: early outcomes from a randomised controlled phase 3 study. Lancet 2016; 388: 1057‐1066. 27474375 10.1016/S0140-6736(16)30592-X

[11] Coughlin GD , Yaxley JW , Chambers SK , et al. Robot‐assisted laparoscopic prostatectomy versus open radical retropubic prostatectomy: 24‐month outcomes from a randomised controlled study. Lancet Oncol 2018; 19: 1051‐1060. 30017351 10.1016/S1470-2045(18)30357-7

[12] Stolzenburg JU , Holze S , Arthanareeswaran VK , et al. Robotic‐assisted versus laparoscopic radical prostatectomy: 12‐month outcomes of the multicentre randomised controlled LAP‐01 trial. Eur Urol Focus 2022; 10.1016/j.euf.2022.02.002 [online ahead of print]. 35216946

[13] Soni PD , Hartman HE , Dess RT , et al. Comparison of population‐based observational studies with randomized trials in oncology. J Clin Oncol 2019; 37: 1209‐1216. 30897037 10.1200/JCO.18.01074 PMC7186578

[14] Jayne D , Pigazzi A , Marshall H , et al. Effect of robotic‐assisted vs conventional laparoscopic surgery on risk of conversion to open laparotomy among patients undergoing resection for rectal cancer: the ROLARR randomized clinical trial. JAMA 2017; 318: 1569‐1580. 29067426 10.1001/jama.2017.7219 PMC5818805

[15] Olavarria OA , Bernardi K , Shah SK , et al. Robotic versus laparoscopic ventral hernia repair: multicenter, blinded randomized controlled trial. BMJ 2020; 370: m2457. 32665218 10.1136/bmj.m2457 PMC7359869

[16] Prabhu AS , Carbonell A , Hope W , et al. Robotic inguinal vs transabdominal laparoscopic inguinal hernia repair: the RIVAL randomized clinical trial. JAMA Surg 2020; 155: 380‐387. 32186683 10.1001/jamasurg.2020.0034 PMC7081145

[17] Ramirez PT , Frumovitz M , Pareja R , et al. Minimally invasive versus abdominal radical hysterectomy for cervical cancer. N Engl J Med 2018; 379: 1895‐1904. 30380365 10.1056/NEJMoa1806395

[18] Lewicki PJ , Basourakos SP , Qiu Y , et al. Effect of a randomized, controlled trial on surgery for cervical cancer. N Engl J Med 2021; 384: 1669‐1671. 33913646 10.1056/NEJMc2035819

[19] Parekh DJ , Reis IM , Castle EP , et al. Robot‐assisted radical cystectomy versus open radical cystectomy in patients with bladder cancer (RAZOR): an open‐label, randomised, phase 3, non‐inferiority trial. Lancet 2018; 391: 2525‐2536. 29976469 10.1016/S0140-6736(18)30996-6

[20] Tan WS , Khetrapal P , Tan WP , et al. Robotic assisted radical cystectomy with extracorporeal urinary diversion does not show a benefit over open radical cystectomy: a systematic review and meta‐analysis of randomised controlled trials. PLoS One 2016; 11: e0166221. 27820855 10.1371/journal.pone.0166221 PMC5098822

[21] Bochner BH , Dalbagni G , Marzouk KH , et al. Randomized trial comparing open radical cystectomy and robot‐assisted laparoscopic radical cystectomy: oncologic outcomes. Eur Urol 2018; 74: 465‐471. 29784190 10.1016/j.eururo.2018.04.030 PMC6697266

[22] NHS England . Clinical commissioning policy: robotic assisted surgery for bladder cancer. 2016. https://www.england.nhs.uk/commissioning/wp‐content/uploads/sites/12/2016/07/16033_FINAL.pdf (viewed Apr 2022).

[23] Catto JWF , Khetrapal P , Ambler G , et al. Robot‐assisted radical cystectomy with intracorporeal urinary diversion versus open radical cystectomy (iROC): protocol for a randomised controlled trial with internal feasibility study. BMJ Open 2018; 8: e020500. 10.1136/bmjopen-2017-020500 PMC6089318 30093510

[24] Catto JWF , Khetrapal P , Ricciardi F , et al. Effect of robot‐assisted radical cystectomy with intracorporeal urinary diversion vs open radical cystectomy on 90‐day morbidity and mortality among patients with bladder cancer: a randomized clinical trial. JAMA 2022; 327: 2092‐2103. 35569079 10.1001/jama.2022.7393 PMC9109000

[25] Deutsch GB , Deneve JL , Al‐Kasspooles MF , et al. Intellectual equipoise and challenges: accruing patients with advanced cancer to a trial randomizing to surgical or nonsurgical management (SWOG S1316). Am J Hosp Palliat Med 2020; 37: 12‐18. 10.1177/1049909119851471 PMC6868288 31122027

[26] McCulloch P , Altman DG , Campbell WB , et al. No surgical innovation without evaluation: the IDEAL recommendations. Lancet 2009; 374: 1105‐1112. 19782876 10.1016/S0140-6736(09)61116-8

[27] Stiller CA . Centralised treatment, entry to trials and survival. Br J Cancer 1994; 70: 352‐362. 8054285 10.1038/bjc.1994.306 PMC2033490

